# Management challenges of perianal necrotizing fasciitis complicated by haemophilia A: A clinical case report

**DOI:** 10.1016/j.ijscr.2023.108470

**Published:** 2023-07-08

**Authors:** Lelai Yu, Ke Yuan, Lina Chen, Yinfu Lei

**Affiliations:** Department of anorectal, Leshan People's Hospital, Leshan 614000, Sichuan Province, China

**Keywords:** Haemophilia, Necrotizing fasciitis

## Abstract

**Introduction and importance:**

Haemophilia A (HA) is a hereditary X-linked recessive hemorrhagic disorder that results from a deficiency or dysfunction of coagulation factor VIII (FVIII) caused by gene mutations.

**Case presentation:**

This case report presents the challenging management of a 37-year-old man who developed perianal necrotizing fasciitis accompanied by severe infection, necrosis, and septic shock. The patient underwent emergency surgery. However, significant bleeding occurred during and after the surgery.

**Clinical discussion:**

Despite initial treatment with fresh frozen blood plasma infusion satisfactory efficacy was not achieved. Investigation into the patient's family history revealed a haemophiliac niece, prompting further testing for haemophilia. Ultimately, the patient was diagnosed with haemophilia A. Hemorrhage controlled was obtained through coagulation factor VIII infusion. With subsequent treatment, the patient experienced significant recovery, and normal anal function was restored.

**Conclusion:**

In summary, routine coagulation examination may not effectively evaluate coagulation dysfunction in patients with severe infectious diseases. Comprehensive preoperative evaluations are necessary for acute anorectal surgeries, with emphasis on screening for haemophilia.

## Highlights

• Perianal necrotizing fasciitis complicated with haemophilia
• Postoperative hemorrhage
• Infusion of coagulation factors to control hemorrhage
• Patient's family medical history

## 1 Introduction

Haemophilia A (HA) is a hereditary X-linked recessive hemorrhagic disorder that results from a deficiency or dysfunction of coagulation factor VIII (FVIII) caused by gene mutations [1]. Clinically, HA is typically manifested as coagulation dysfunction, excessive bleeding after trauma, or spontaneous bleeding. Perianal necrotizing fasciitis is a severe infectious disease characterized by progressive fascial necrosis, which is an acute and critical condition in the anorectal surgery department and requires extensive debridement in an emergency [2]. However, the case of perianal necrotizing fasciitis combined with haemophilia is rarely reported. If haemophilia escapes diagnosis preoperatively, the operative risk will be exacerbated dramatically. The diagnosis and treatment of a case of HA complicated with perianal necrotizing fasciitis admitted to our department are reported as follows.

## 2 Presentation of case

A 37-year-old male patient presented to our department due to a one-week history of severe perianal swelling and pain. Physical examination indicated a body temperature (T) of 39.6 °C, a pulse (P) of 127 beats/min, a respiratory rate (R) of 20 breaths/min, blood pressure (BP) of 97/53 mmHg, and a body weight of 80 kg. Anorectal examination revealed a 20x20cm^2^ redness and swollen area around the anus and buttocks. The redness and swollen area affected the perineum on the anterior side of the anus, causing diffuse swelling of the lower edge of the scrotum. The swollen area had subcutaneous crepitus, and the local skin was dark brown. For laboratory test results, blood routine: white blood cell count (WBC) of 15.36 × 10^9^/L, neutrophil count (NEUT#) of 12.88 × 10^9^/L, neutrophil percentage (NEUT%) of 83.9 %, and high sensitivity C-reactive protein (hsCRP) of 248.27 mg; Five coagulation tests: prothrombin time (PT) of 14.1 s, international normalized ratio (INR) of 1.11, activated partial prothrombin time (aTPP) of 59.9 s, fibrinogen (FIB) of 7.85 g/L and thrombin time (TT) of 15.9 s; liver and kidney function: alanine aminotrasferase (ALT) of 199 U/L, aspartate aminotrasferase (AST) of 55 U/L, total protein (TP) of 63.2 g/L, and albumin (ALB) of 36.2 g/L. Admission diagnosis: perianal necrotizing fasciitis.

**Fig. 1:**
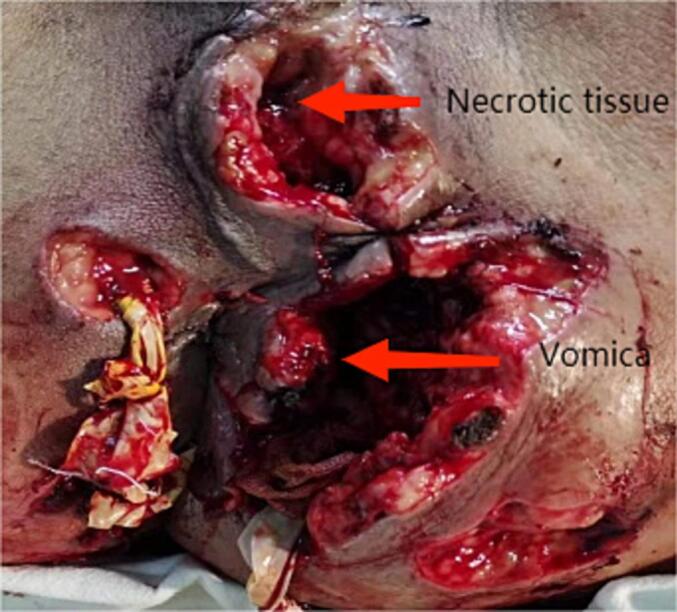
After the first debridement.

**Fig. 2:**
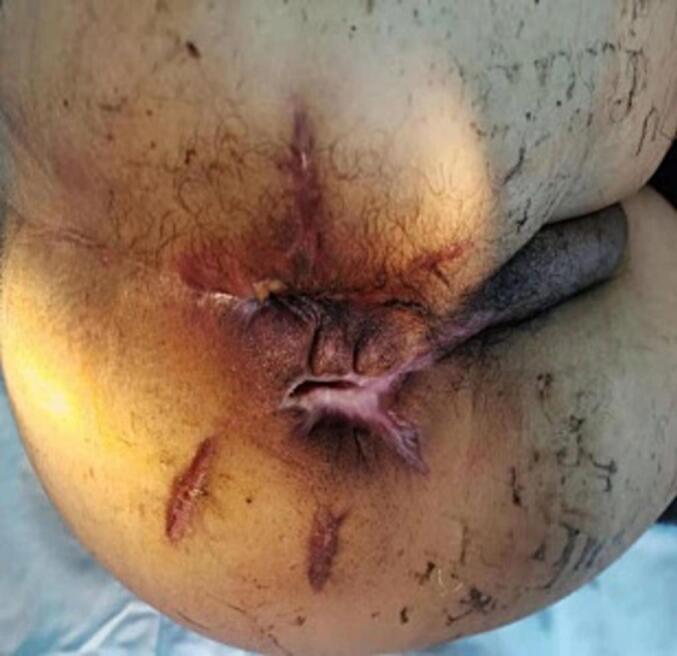
Complex anal fistula 2 months after surgery.

**Fig. 3:**
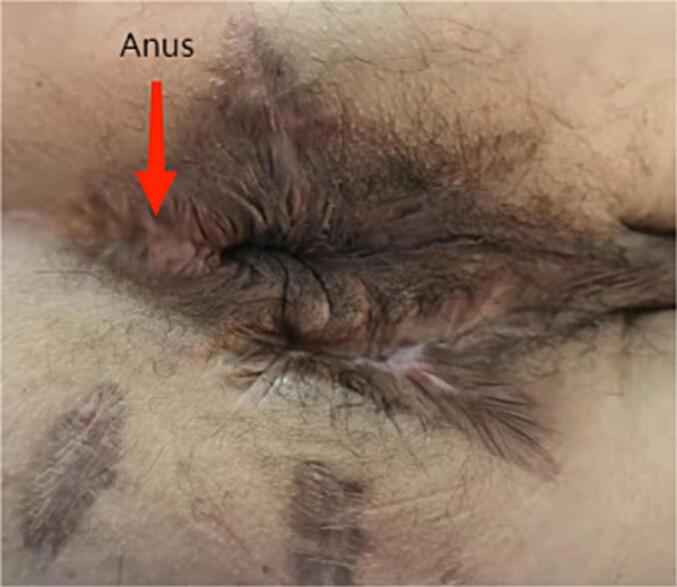
Reexamination 1 year after surgery.

Under general anesthesia, necrotizing fasciitis wound reconstruction and drainage + multiple incision drainage for abscesses were performed in the emergency. During the surgery, there was significant bleeding on the wound surface, with approximately 100 mL of intraoperative bleeding. The wound surface and abscess cavity were treated with vaseline gauze packing along with hemostasis by compression bandage. The condition after debridement is shown in Fig. 1. After surgery, the patient developed drowsiness, poor mental state, and low blood pressure (fluctuations of 70–80/50–60 mmHg). He was then transferred to an intensive care unit due to suspicions of septic shock. The patient received anti-infectious therapy with meropenem and vancomycin, and underwent a blood transfusion to correct hemorrhagic anemia and supplement albumin. However, the wound dressing exhibited obvious bleeding in the absence of pressure. Subsequently, red blood cell suspension and fresh frozen plasma were administered respectively to correct anemia and improve coagulation dysfunction. After inquiring about the patient's family medical history, it was found that his niece was clearly diagnosed with HA. Hence, coagulation factor-related tests were further performed, and the activity of coagulation factor VIII was found to be 4.4 % (reference value 60–150 %), confirming the diagnosis of HA (Table 1). Accordingly, the patient was subjected to coagulation factor VIII infusion, with an infusion dosage and course of 1600Uxq12hx7 days. After the first infusion of coagulation factor VIII, there was a significant improvement in wound bleeding. Further perianal magnetic resonance imaging suggested a high complex anal fistula. Transanal Opening of Intersphincteric Space (TROPIS) procedure combined with Parks incision-thread-drawing was performed under general anesthesia, and the patient was discharged after recovery (Fig. 2). After 1 year of surgery, the anal function was reexamined, with a Wexner score of 0 and normal control function of dry stool, gas, and fluid. The appearance of the anus is shown in Fig. 3. The work has been reported in line with the SCARE criteria [3].

**Table 1:** The diagnosis of HA.

Report in Department of Laboratory Medicine, West China Hospital of Sichuan University					
Name: Tong Yichuan	Sex: Male	Age: 37	No:20200420G004-0802	Dept: Neurology Department	
Sample: Plasma	Case No:0014144875	Diagnosis: Coagulation disorder			
No	Items	Results		Unit	Reference value
1	Multipoint dilution VIII (1:1) (MDA(1/1))	24.50	↓	%	60–150
2	Multipoint dilution VIII (1:2) (MDA(1/2))	26.70	↓	%	60–150
3	Multipoint dilution VIII (1:4) (MDA(1/4))	26.30	↓	%	60–150
4	Activity of factor VIII (F VIII:C)	25.80	↓	%	60–150

## 3 Discussion

During the first surgery, there was obvious bleeding on the wound surface. It was considered that the bleeding was attributed to coagulation dysfunction caused by infection and a large necrosis range. The patient experienced continuous wound bleeding after the surgery. After a detailed inquiry of family medical history and implementation of coagulation factor examination, the patient was definitely diagnosed with HA.

Haemophilia has a high tendency of missed diagnosis due to its extremely low incidence rate and difficulty in early diagnosis. APTT prolongation is common in coagulation disorders caused by insufficient endogenous coagulation factors [ [4]]. The preoperative APTT of this patient was 59.9 s, exceeding 10 s. The preoperative evaluation indicated coagulation dysfunction caused by severe infection, and the medical history inquiry was not detailed enough, resulting in missed diagnosis of haemophilia before surgery. The main principle of haemophilia treatment is to prevent and treat bleeding and bleeding-related complications. Alternative therapy, which involves supplementing missing coagulation factors, is currently the most effective treatment method for haemophilia. The administration of fresh frozen plasma and cryoprecipitate before diagnosis in this case played a significant clinical role, avoiding the possibility of life-threatening bleeding.

Conducting a comprehensive hematological workup in young patients with rare clinical manifestations offers several benefits. Key considerations include: identifying previously undiagnosed hematological conditions that may be contributing to the rare presentation, providing essential information for formulating a customized treatment plan, facilitating the detection of any associated coexisting conditions for comprehensive management and timely interventions, and identifying potential genetic factors to offer genetic counseling to patients and their families.

## 4 Conclusion

In conclusion, there are few reports on haemophilia complicated with perianal diseases currently. Especially for patients complicated with severe infectious diseases, it is difficult to evaluate the coagulation dysfunction only by routine coagulation examination before surgery. It is crucial to revisit and reassess the initial diagnosis, adjusting it based on the patient's specific condition. What's more, it is necessary to do a full preoperative evaluation before acute and severe anorectal surgery, attach importance to the preoperative screening of haemophilia, and further improve the perioperative risk assessment and standardized treatment of haemophilia.

## Ethical approval

Ethical approval for this study (Ethical Committee N°LYLL 021) was provided by the Ethical Committee Leshan People's Hospital review board, Sichuan, China on 23 March 2023.

## Funding

This research did not receive any specific grant from funding agencies in the public, commercial, or not-for-profit sectors.

## Author contribution

**Lelai Yu**: Data curation; Formal analysis; Roles/Writing - original draft; Writing - review & editing.

**Ke Yuan**: Data curation; Writing - review & editing.

**Lina Chen**: Formal analysis; Writing - review & editing.

**Yinfu Lei**: Conceptualization; Writing - review & editing.

## Guarantor

Yinfu Lei.

## Research registration number

Not applicable.

## Consent

Written informed consent was obtained from the patient for publication of this case report and accompanying images. A copy of the written consent form is available for review by the Editor-in-Chief of this journal on request.

## Conflict of interest statement

The authors declare that they have no conflicts of interest.
